# Robot steering-angle prediction lightweight network based non-local attention and lane guidance

**DOI:** 10.1371/journal.pone.0339409

**Published:** 2026-01-20

**Authors:** Jing Niu, Jiahao Zheng, Chuanyan Shen, Guanghao Gao, Guoqiang Song, Shifeng Liu, Yibo Wang

**Affiliations:** 1 School of Mechatronics and Automotive Engineering, Tianshui Normal University, Tianshui, China; 2 Key Laboratory of Resource Utilization of Agricultural Solid Waste in Gansu Province, Tianshui Normal University, Tianshui, China; Firat University, TÜRKIYE

## Abstract

Predicting the steering angle of robots is a core challenge in autonomous navigation. This paper proposes a novel end-to-end prediction network that integrates non-local attention and lane line guidance mechanisms to significantly reduce computational time and improve prediction accuracy. Built upon the ResNet architecture, the network incorporates a Non Local Block mechanism to enhance global context modeling and a Ghost Module to reduce parameter count and improve feature extraction efficiency. To further optimize training, a ReduceLROnPlateau learning rate scheduler is employed to adaptively adjust the learning rate, effectively mitigating overfitting. Additionally, a lane line annotation method, which combines Canny edge detection with the Hough transform, is used to semantically guide the input images. This enhances the representational power and generalizability of the training data. Experimental results demonstrate that the proposed network outperforms the baseline across multiple evaluation metrics. Under identical experimental conditions, the proposed model achieves a 54.88% increase in inference speed and reduces the mean absolute error (MAE) and root mean square error (RMSE) by 8.47% and 18.23% respectively. Ablation studies further confirm that the combination of the Non-Local Block and Ghost Module significantly improves both expressive capacity and computational efficiency of the model. These findings suggest that the proposed method offers a high-precision, efficient, and low-latency visual perception solution for real-time autonomous navigation of wheeled robots in complex environments.

## 1. Introduction

With the rapid advancement of artificial intelligence, wheeled robots are increasingly applied in warehouse logistics, intelligent transportation, and emergency rescue. Auto-navigation is a core technology that enables these robots to operate intelligently. During navigation, wheeled robots rely on high-precision environmental perception and path planning to drive safely and avoid obstacles. Among these capabilities, steering angle-prediction plays a pivotal role, as it directly influences both path planning and obstacle avoidance. However, traditional methods, which depend on expert-designed features followed by encoding and fusion to construct object detection models, perform poorly in complex and dynamic environments [1], limiting their practical effectiveness. Recent progress in end-to-end autonomous driving has shown promise in addressing these limitations. One notable example is the PilotNet model proposed by Nvidia in 2017 [2], which uses convolutional neural networks (CNNs) to directly learn the mapping from input images to steering angles [3]. Visualization tools have demonstrated PilotNet’s ability to automatically recognize key driving features such as lane lines and road edges. However, because it relies solely on static information from individual frames, it cannot model the continuity of driving behavior, limiting its performance in dynamic environments. Real-world driving scenarios involve significant spatiotemporal dependencies, making it difficult to achieve accurate control using spatial features alone. Therefore, integrating temporal and spatial features has become essential for improving the performance of autonomous driving models. In response, researchers have proposed several end-to-end frameworks that address this need. For example, the University of California developed an FCN-LSTM architecture that combines fully convolutional networks (FCNs) with long short-term memory networks (LSTM) [4]. This approach extracts spatial features from individual frames using FCNs and fuses temporal information from historical frames through LSTMs to output more accurate control commands. It also incorporates image segmentation tasks to improve scene understanding [5]. Chen et al. trained an AlexNet-based model on large-scale simulated driving datasets and achieved automated driving on multilane highways [6]. However, due to the lack of temporal modeling, its performance was unstable under complex road conditions. Although Nvidia’s follow-up research demonstrated success in real-world tests, it still underutilized sequential frame information, limiting the accuracy of control prediction [7]. Deep reinforcement learning has also been explored in this domain. For instance, Mobileye applied the policy gradient iteration method to derive optimal driving policies [8], while El Sallab et al. employed the deep deterministic policy gradient (DDPG) algorithm to train agents in the open-source TORCS driving simulator [9]. Despite their promising results in simulations, these methods still face challenges such as limited prediction accuracy, high model complexity, and redundancy in extracted features.

The primary reason for insufficient prediction accuracy in existing models is their inability to efficiently capture long-range dependencies in visual scenes. Capturing such dependencies is essential for deep neural networks; however, traditional solutions such as deep convolutional networks rely on repetitive local operations, resulting in low computational efficiency and challenging optimization. The Non-local Block proposed by Wang addresses this issue by modeling feature information across long spatial distances and capturing remote dependencies [10]. This component is efficient, simple, and broadly applicable, as it computes the response at any location as the weighted sum of features from all locations. Gkioxari et al. demonstrated its effectiveness by introducing the Non-local Block into a network branch for interaction classification, enabling the model to better capture remote spatial and semantic relationships between humans and objects and to more accurately predict interaction behaviors [11]. Another major challenge in deploying deep learning models on embedded platforms with limited computing resources is high model complexity and feature redundancy. To address this, Han et al. proposed the Ghost Module, which generates additional feature maps using low-cost operations [12]. This module significantly reduces parameter count and computational load while maintaining accuracy [13]. Its core principle is to minimize computational cost and the number of parameters while preserving feature richness. The strong performance of the Ghost module has been verified in the GhostNet architecture, where it serves as a foundational building block [12]. Building on this, Tang et al. developed GhostNetV2, which demonstrated that architectures centered on the Ghost Module can achieve an excellent balance between accuracy, FLOPs, and inference speed across key visual tasks such as image classification and object detection [14]. These studies collectively highlight the Ghost Module’s strong potential as a lightweight core component.

Lightweight architectures based on attention mechanisms have recently become a significant research focus in mobile robot navigation. For example, the PTPO framework proposed by Zhang et al. dynamically optimizes sense-driven ratios through neural learning, substantially improving path tracking accuracy [15]. Inspired by this work, the present study innovatively integrates the Non-local Block with the Ghost Module to develop a steering angle prediction network that combines non-local attention and lane line guidance. By leveraging the complementary strengths of these two modules, the proposed approach addresses both accuracy and complexity in steering Angle prediction. Specifically, the Non-local Block and Ghost Module are incorporated into a ResNet18 backbone to enhance the model’s ability to capture global contextual information while reducing computational complexity. In addition, the ReduceLROnPlateau module is introduced into the training framework to dynamically adjust the learning rate, thereby improving model stability and accuracy. The collected training set is further diversified and enhanced using runway marking operations with Canny edge detection and the Hough transform, which provide explicit feature information and help the model better capture key features. Experimental results show that the proposed network offers significant improvements in prediction accuracy and operational efficiency, greatly enhancing the end-to-end autonomous navigation performance of wheeled robots in complex scenarios.

## 2. Related work

### 2.1. End-to-end networks for autonomous driving scene learning

**Fig 1:**
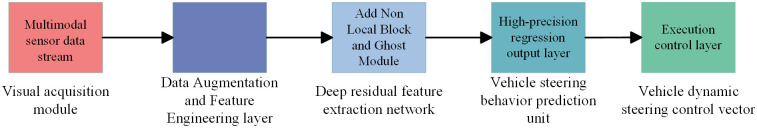
The end-to-end learning network architecture for robot auto-navigation.

End-to-end learning offers significant advantages in autonomous navigation tasks for wheeled robots. This approach enables direct extraction of decision-making logic from collected operational data, resulting in stronger adaptability and generalization capabilities [16]. By inputting image data from onboard sensors into deep learning algorithms, particularly CNNs [17], the model can automatically learn informative features and predict steering angles. This eliminates the need for complex manual feature engineering [18]. The core idea of end-to-end learning is to train a single neural network model to directly map raw input data (such as images, radar signals, LiDAR signals) to control commands (such as steering, acceleration, or braking). This paradigm emulates the intuitive behavior of human drivers, who perceive and respond to their environment without relying on intermediate steps [19]. The steering angle prediction network proposed in this study follows this architecture, as illustrated in Fig 1. It maps camera images to predicted steering angles through a unified neural network.

Traditional CNNs often struggle with complex visual tasks due to their limited capacity to capture long-range spatial dependencies and their constrained feature representations. To overcome these limitations, this paper introduces an enhanced ResNet-based deep residual network that incorporates non-local attention and lane line guidance. This architecture enables direct mapping from driving scene images to steering angle predictions, improving both prediction accuracy and computational efficiency.

### 2.2. Non-Local Block module

The Non-Local Block is a neural network component designed to capture dependencies across all positions in a feature map, making it well-suited for modeling global context. Unlike traditional CNNs that rely on local operations with limited receptive fields, Non-Local Block can model long-range spatial relationships and improve performance in tasks such as object detection, image classification, and generation [20]. This capability is particularly valuable in autonomous driving, where visual inputs are complex and contain widely distributed contextual cues. Accordingly, the Non-Local Block is integrated into the ResNet18 baseline network in this study. The Non-Local Block computes a new output for each position by calculating a weighted sum of the features from all other positions in the input. This operation can be mathematically represented as:


$$y_{i}=\frac{\text{1}}{C_{x}}\sum_{\forall j}f\left(x_{i},x_{j}\right)g\left(x_{i}\right)$$ (1)

In the formula, X is the input feature map, representing either raw pixel data or intermediate outputs from a neural network layer; $f\left(x_{i},x_{j}\right)$ is a similarity function used to measure the relationship between positions $x_{i}$ and $x_{j}$ in the input signal; $g\left(x_{i}\right)$ is the transformed feature at position $x_{i}$; $C_{x}$ is the normalization factor; $y_{i}$ is the output signal, which is calculated by taking a weighted average over all positions in the input using the similarity scores. It is the updated value of the input signal $x_{i}$ after the Non-Local Block operation [10].

**Fig 2:**
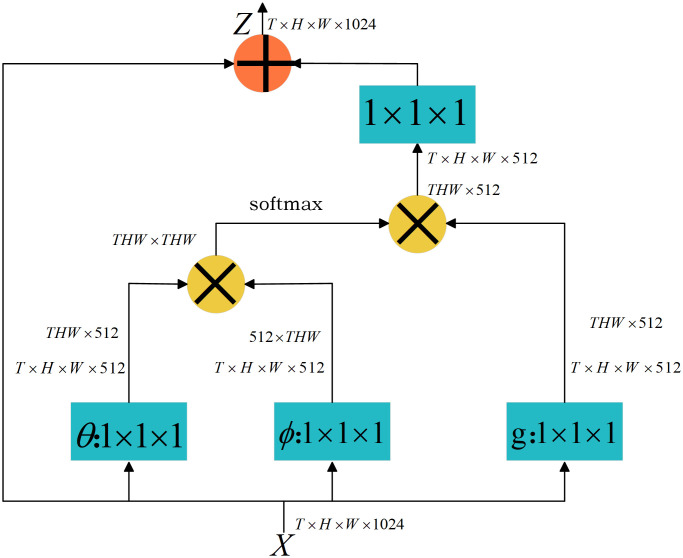
Non-Local Block module structure.

In Equation (1), for each position $x_{i}$ in the image, the similarity with all other positions $x_{j}$ is calculated (using a function f), and then the features of the other positions are weighted and normalized (using a function $g\left(x_{i}\right)$) to obtain the updated output $y_{i}$. This operation establishes global dependencies and allows the model to capture contextual relationships throughout the entire image. The structural design of the Non-Local Block is depicted in Fig 2.

In Fig 2:

**Input** X: The feature map has dimensions T×H×W×1024 , where T is the number of frames (or temporal steps), H×W is the spatial dimension of the feature map, and 1024 is the number of channels.

**Branch** θ: Applies a 1×1 convolution to reduce the number of channels to 512, producing an output of size T×H×W×512.

**Branch** ϕ: Applies the same 1×1 convolution as Branch θ, resulting in a matching output of size T×H×W×512.

**Branch** g: Applies a 1×1 convolution to produce a reduced-dimensional representation for use in the attention mechanism, with output size T×H×W×512.

***Softmax***: Computes pairwise similarity scores by performing matrix multiplication between the outputs of Branch θ and Branch ϕ. These scores are normalized with a Softmax function to generate the attention weight matrix. The output is THW×THW which represents the similarity between each location in the input feature map.

**Feature Aggregation**: The attention weights are used to scale the output from Branch g, generating a globally weighted feature map with size T×H×W×512.

**Output Z**: A final 1×1 convolution restores the channel dimension to 1024. A residual connection adds the original input to the weighted feature map, yielding the final output with size T×H×W×1024.

### 2.3. Ghost Module

The Ghost Module is a lightweight neural network component designed to generate a large number of feature maps using minimal computation. It enables efficient feature representation while significantly reducing the computational and parameter complexity of the model [21]. Integrating this module into the network greatly lowers the time cost of model inference without compromising representational power.

**Fig 3:**
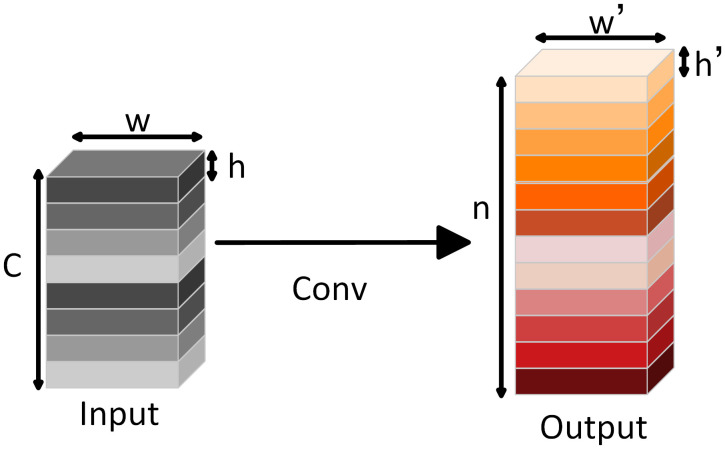
Traditional convolutional layer structure.

**Fig 4:**
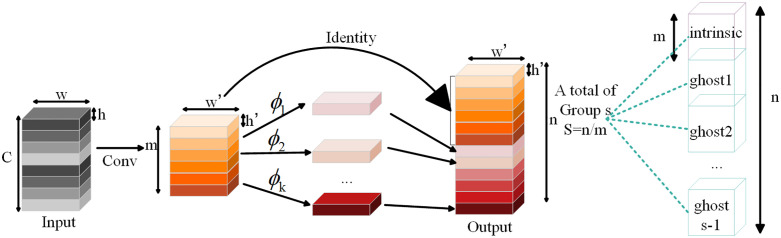
Ghost Module structure.

Traditional convolutional layers rely on extensive convolution operations to produce high-dimensional feature maps, which leads to substantial computational overhead. In contrast, the Ghost Module reduces this load by performing only a subset of full convolutions. Specifically, half of the feature maps are generated through standard convolution, while the remaining are produced via simple linear transformations applied to the initial set [22]. Additionally, the module adopts depthwise separable convolutions to extract redundant information, further reducing computational overhead [12]. This dual strategy ensures that the model maintains expressive capacity while minimizing unnecessary processing. The difference between traditional convolutional layers (illustrated in Fig 3) and the Ghost Module (shown in Fig 4) can be summarized as follows: In traditional convolution, an input feature map is convolved using multiple kernels to produce the output feature map, which requires heavy computation. In the Ghost Module, a smaller number of base feature maps are first generated using full convolutions with m kernel operations. These base maps are then transformed through inexpensive linear operations to generate additional “ghost” feature maps. The final output consists of both the original base features and their corresponding ghost maps.

Unlike traditional convolution that generates all output maps through costly operations, the Ghost Module creates new feature maps using lightweight transformations, ensuring efficient information representation [23]. In Fig 4, Identity refers to the original input feature map, which serves as the foundational input and is preserved during processing. The subsequent layers generate additional maps through convolutions and transformations. These ghost features are combined with the original input to form the complete output, providing both efficiency and completeness. The computational efficiency of the Ghost Module relative to traditional convolution can be quantified using FLOPs (floating point operations), a common metric for measuring algorithmic complexity [24]. The theoretical formulations are as follows:


$$FLOPs=C_{out}\times H_{out}\times W_{out}\times C_{in}\times K\times K$$ (2)

For traditional convolutional layer structures:


$$FLOPs_{1}=n\times h^{\prime }\times w^{\prime }\times c\times k\times k$$ (3)

For the intrinsic part in the Ghost Module:


$$FLOPs_{2}=n/s\times h^{\prime }\times w^{\prime }\times c\times k\times k$$ (4)

For the ghost feature map section in the Ghost Module:


$$FLOPs_{3}=(s-1)\times h^{\prime }\times w^{\prime }\times n/s\times d\times d$$ (5)

For the entire Ghost Module structure:


$$FLOPs_{4}=FLOPs_{2}+FLOPs_{3}$$ (6)

Then there are:


$$FLOPs_{1}÷FLOPs_{4}\approx s÷\left[1+\left(s-1\right)/c\right]\approx s$$ (7)

In the formula: k×k is the size of the kernel in traditional convolution structures, the first d is the number of input channels for linear transformation, the second d is the number of output channels for linear transformation, m=n/s, s is the number of ghost feature maps generated per base feature map (typically small), and c denotes the number of input channels, which is generally large [25]. These equations validate that the Ghost Module provides a substantial reduction in computational complexity while retaining robust feature representation, making it highly suitable for real-time, resource-constrained robotic applications.

## 3. Lightweight prediction network based on Non-Local Block and Ghost Module

### 3.1. ResNet as the base network

While Non-Local Block and Ghost Module are effective for complex visual tasks, their performance depends on a robust backbone network architecture. In CNNs, convolutional layers are the core components for feature extraction. Although deeper networks theoretically provide stronger feature extraction capabilities, increasing depth often introduces challenges such as vanishing gradients and network degradation. To address these issues, He et al. proposed the Residual Network (ResNet) model, which incorporates skip connections to improve the stability of deep network training [26]. Among the ResNet family, ResNet18 is a popular choice for lightweight applications due to its compact architecture, reduced parameter count, and low computational complexity [27]. Compared with deeper variants, ResNet18 offers faster training, simpler hyperparameter tuning, and better suitability for rapid deployment in real-world scenarios [28]. Accordingly, this study adopts ResNet18 as the base architecture for further enhancement.

ResNet18 effectively solves the problem of gradient vanishing while maintaining deep representation learning through its residual structure. By utilizing both its depth and residual connections, it provides a strong foundation for integrating the Non-Local Block and Ghost Module to achieve more accurate feature learning and efficient computation.

**Fig 5:**
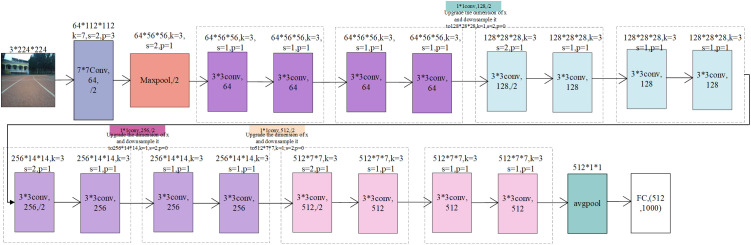
ResNet18 baseline network architecture. Conv is convolution; s is the step size; k is the size of the convolution kernel; p is for filling; Maxpool is the pooling of the maximum value; avgpool is average pooling.

The adopted ResNet18 baseline network includes a 7 × 7 convolutional layer for initial feature extraction, followed by 16 3 × 3 convolutional layers (arranged as 8 residual blocks, each with two convolutional layers) [29]. Finally, a fully connected layer maps the learned features to the output space for regression-based steering angle prediction. This architecture effectively captures both local and global image features, enabling accurate decision-making in autonomous navigation. The network structure is illustrated in Fig 5.

### 3.2. Improved ResNet18 with Non-Local Block and Ghost Module

**Fig 6:**
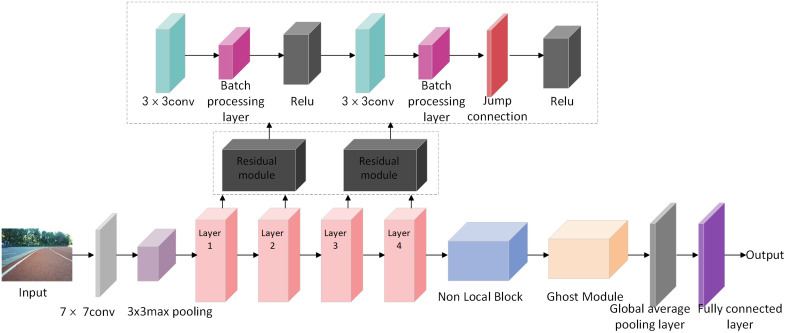
The improved ResNet18 network architecture integrates the Non-Local Block and Ghost Module.

Although ResNet18 performs well in visual feature extraction, it still faces limitations in capturing global contextual information and optimizing computational efficiency in complex, dynamic environments. To address these gaps, this paper introduces an improved network architecture that incorporates both the Non-Local Block and Ghost Module into ResNet18. The overall network structure is shown in Fig 6.

Specifically, the input is a depth image of the robot’s operational environment. Initial features are extracted using a 7 × 7 convolutional layer, followed by a 3 × 3 max pooling layer to reduce the spatial resolution of the feature map. The residual module comprises four stages (Layer1 to Layer4), each containing multiple residual blocks. Each residual block includes two 3x3 convolutional layers, followed by a batch normalization layer and a ReLU activation. Skip connections are applied to add the input directly to the output, forming the residual structure.

A Non-Local Block is inserted after Layer 4 to capture long-range dependencies. It computes attention weights based on feature similarity and applies them across the feature map to model global relationships without changing the output dimensions. This improves the model’s ability to interpret complex spatial interactions. Following the Non-Local Block, a Ghost Module was added to further reduce parameter count and computational load. It generates ghost feature maps using a combination of convolutions and lightweight depthwise separable convolutions. This operation retains the same output dimensions while making the model more efficient and better suited for embedded systems with limited resources. The output of the Ghost Module is passed through a global average pooling layer, which compresses the spatial dimensions. A fully connected layer then flattens the pooled features and performs the final regression task to predict the steering angle. This improved design retains the benefits of ResNet18’s residual connections while enhancing the model’s robustness and real-time performance through global context modeling and lightweight computation.

## 4. ReduceLROnPlateau for adaptive learning rate

### 4.1. Training with a static learning rate

**Fig 7:**
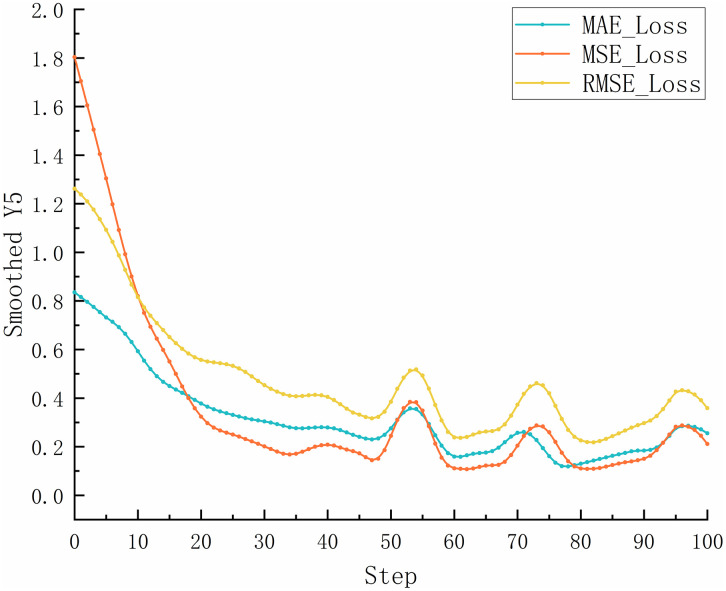
The variation curves of MAE, MSE, and RMSE for the static learning rate as $1\times 10^{-4}$.

**Fig 8:**
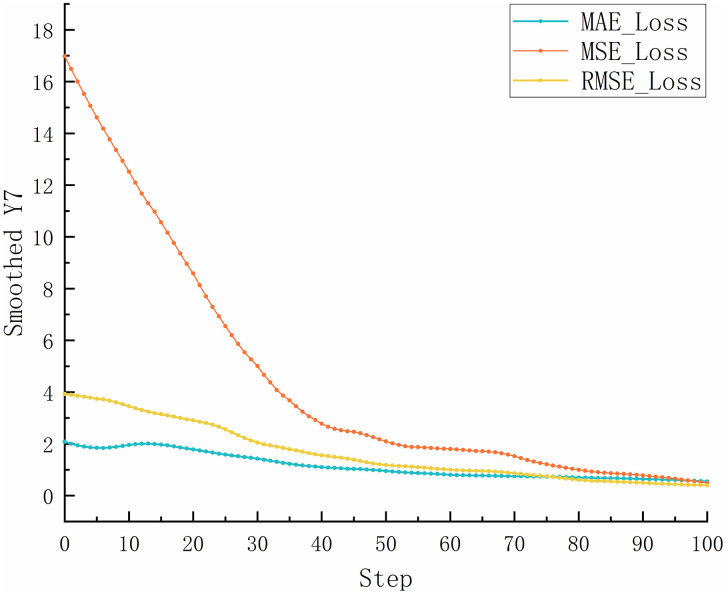
The variation curves of MAE, MSE, and RMSE for the static learning rate as $1\times 10^{-6}$.

In conventional neural network training, static learning rates are commonly used. However, fixed learning rates can be inadequate when handling complex and highly variable datasets [30]. Selecting an appropriate static learning rate is also a challenge. If set too high, the learning rate can cause large fluctuations in the loss function during training, which leads to unstable model performance [31]. Conversely, setting it too low may slow convergence and increase the risk of overfitting due to insufficient gradient update steps [32,33]. The test results using a static learning rate of $\text{1}\times {\text{10}}^{-\text{4}}$ for the proposed prediction network are shown in Fig 7. As illustrated, a high learning rate causes the model to converge rapidly but results in severe oscillations in the loss curve. This indicates that the step size for parameter updates is too large, which causes the optimizer to overshoot the optimal solution repeatedly. As a result, the model fails to converge stably, significantly reducing its generalization ability and producing unstable predictions in real-world applications. Fig 8 shows the test results when the learning rate is reduced to $\text{1}\times {\text{1}\text{0}}^{-\text{6}}$. In this case, the training error decreases extremely slowly, and the loss curve exhibits a nearly linear decline. The insufficient step size for gradient updates prevents effective parameter optimization. This results in underfitting and poor model performance.

### 4.2. Dynamic learning rate strategy

To improve model stability and performance, this study introduces a dynamic learning rate adjustment strategy. Given the potential for validation error to plateau or oscillate during training, an adaptive mechanism based on validation loss is adopted. This is the ReduceLROnPlateau strategy. This scheduler monitors the validation loss for the t-th round $\lambda_{t}$, and the current learning rate is $\eta_{t}$. The adjustment strategy is as follows:


$$\eta_{t+1}=\{\begin{array}{c} {}*20l\eta_{t}\;\;\;\;\;\;\;\;\;\;\;\;\;\;\;\;\;\;\;\;\;\;\;\;\;\;\;\;\;\;\;\;\;\;if\;\lambda_{t}\leq min\left(\lambda_{t-p},......\lambda_{t}\right)\\ \gamma \cdot \eta_{t}\;\;\;\;\;\;\;\;\;\;\;\;\;\;\;\;\;\;\;\;if\;loss\;does\;not\;improve\;within\;p\;consecutive\;epochs \end{array}$$ (8)

Among them γ∈(0,1) is the learning rate decay factor (set to 0.5), and P is the patience parameter, which refers to the number of consecutive epochs without improvement in validation loss (set to 2 in this study). In this study, it is: $\eta_{t+1}$ is the next learning rate. When the validation loss fails to improve over two consecutive epochs, the learning rate is reduced to half its current value. This strategy enhances training stability and accelerates convergence [34]. Unlike fixed-interval schedulers, ReduceLROnPlateau does not require a manually defined adjustment schedule. This flexibility makes it particularly suitable for dynamically changing tasks such as steering angle prediction.

**Fig 9:**
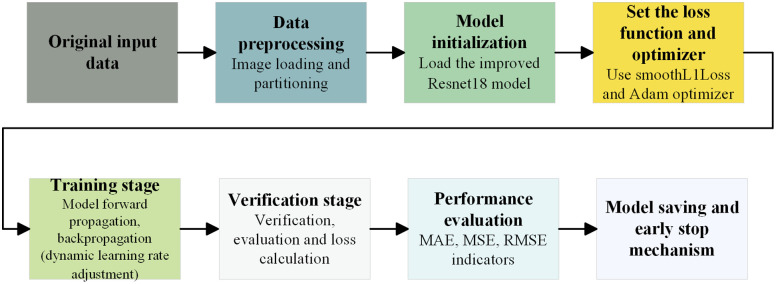
ReduceLROnPlateau implements an adaptive learning rate process.

The full training pipeline with ReduceLROnPlateau is illustrated in Fig 9. First, the input image is resized to 120ⅹ160 pixels, converted to a tensor, and normalized. The dataset is then split into a training set and a validation set, with shuffling applied only to the training set. A custom network is used, with SmoothL1Loss as the loss function and the Adam optimizer initialized with a learning rate of $\text{3}\times {\text{1}\text{0}}^{-\text{4}}$. The ReduceLROnPlateau scheduler monitors validation loss and reduces the learning rate by 50% if there is no improvement over two consecutive epochs. During each training epoch, the model performs forward propagation, loss computation, backpropagation, and parameter updates. The training performance is evaluated using metrics such as mean absolute error (MAE), mean square error (MSE), and root mean square error (RMSE). During validation, gradients are disabled to prevent parameter updates. The model is monitored using a validation loss-based checkpoint mechanism. If the current validation loss is lower than the best recorded value, the model weights are saved. If the validation loss fails to improve over five consecutive epochs, early stopping is triggered to terminate training and avoid overfitting.

## 5. Experiments and analysis

### 5.1. Equipment and data

**Fig 10:**
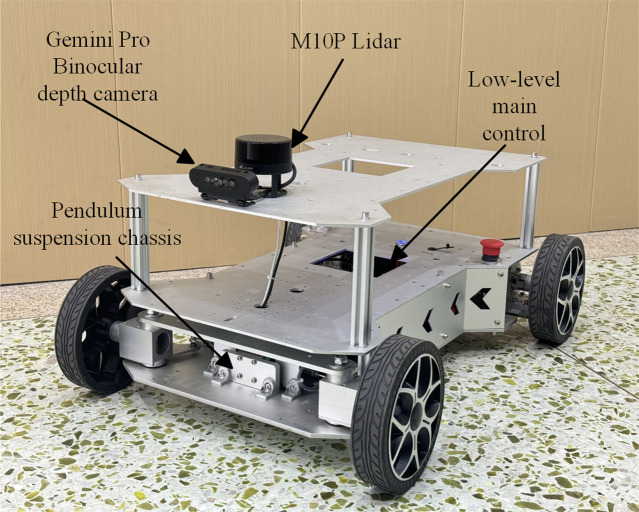
The structure of wheeled robots.

The image dataset used in this experiment was collected from runway environments under various weather conditions. The structural setup of the wheeled robot is illustrated in Fig 10. The robot is equipped with an Obi Zhongguang Gemini Pro dual-lens depth camera, a coaxial pendulum suspension system, a low-level main control unit, and an M10P LiDAR. The depth camera has a horizontal field of view of 71.5°, a vertical field of view of 56.7°, and supports a depth sensing range from 0.25 meters to infinity. It operates at a maximum frame rate of 30frames per second and a resolution of 1920 × 1080 pixels. The robot has a maximum movement speed of 0.63 meters per second.

**Fig 11:**
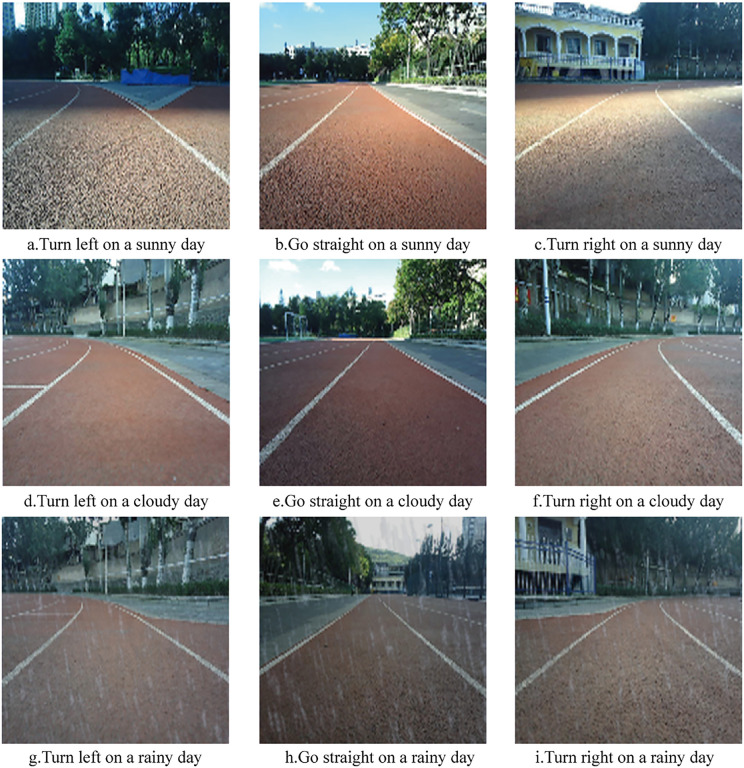
Example of the dataset image.

The dataset was captured at a frequency of 12 frames per second. During data collection, each image file was automatically labeled with the robot’s current steering angle. To accommodate varying weather conditions (sunny, cloudy, rainy) and motion states (left turn, straight go, right turn), the dataset was organized into nine folders. In total, 23,478 images were collected. Fig 11 displays representative images of left turns, straight movement, and right turns under each weather condition.

The dataset was randomly divided into training, validation, and test sets using a 7:2:1 ratio. A detailed distribution of image counts across these subsets is provided in Table 1. Only the images in the training set were subjected to further preprocessing.

**Table 1:** Distribution of image quantity in the dataset.

Turn	Training set	Validation set	Test set	Total
Turn left	4680	1337	668	6685
Go straight	5541	1583	793	7917
Turn right	6213	1776	887	8876
Total	16434	4696	2348	23478

### 5.2. Dataset enhancement process

**Fig 12:**
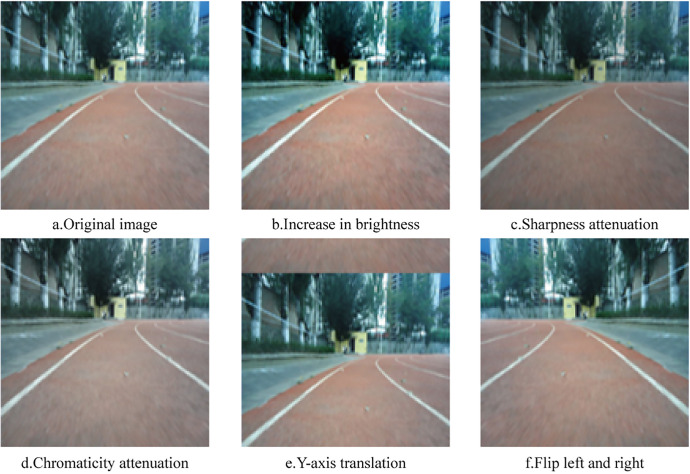
Example of dataset preprocessing.

To improve the accuracy and robustness of the prediction network, several image enhancement techniques were applied to diversify the training data set. These techniques included adjustments in contrast, brightness, sharpness, and chromaticity, as well as geometric transformations such as image translation and horizontal flipping. Representative examples of these augmentations are shown in Fig 12.

### 5.3. Canny edge detection and Hough transform for runway enhancement guidance

Image processing algorithms are essential components of machine vision applications, with edge detection playing a particularly critical role. Edge detection highlights key regions in an image, making it vital for downstream processing tasks [35]. In this study, in addition to conventional data augmentations such as brightness and chromaticity adjustments, and geometric flips (up, down, left, right), the Canny Edge Detection algorithm, widely recognized for its superior edge detection performance, is employed to extract runway features and mark runway boundaries. The classic Canny edge detection process consists of the following steps [36]:

(1) Gaussian filtering: A Gaussian filter is applied to the original image to suppress noise and smooth the image.
(2) Gradient Calculation: Gradient amplitude and directions are computed in four directions (0 °, 45 °, 90 °, 135 °) using operators such as Roberts, Prewitt, and Sobel [37]. The gradient magnitudes and directions are calculated from horizontal and vertical differences, as follows:

$$G=\sqrt{{G_{x}}^{2}+{G_{y}}^{2}}$$ (9)

$$\theta =\arctan\left({G_{y}}^{2}÷{G_{x}}^{2}\right)$$ (10)

(3) Non-Maximum Suppression: Only the maximum gradient values along the gradient direction are retained, while non-maximum values are suppressed to refine edge contours.
(4) Dual-Thresholding and Edge Tracking: Unlike single threshold methods, Canny edge detection uses both high and low thresholds to distinguish edge pixels. Pixels with gradients above the high threshold are marked as strong edges, those below the low threshold are discarded, and pixels falling between the two are classified as weak edges, which are retained only if connected to strong edges [38–40].

After extracting complete runway edges using Canny detection, the Hough Transform is applied to detect straight lines. The Hough transform is a classic technique in image processing for identifying the parameters of geometric shapes such as lines and circles [41,42]. The algorithm works by converting the image space into a parameter space, where the detection of line parameters becomes a peak-finding problem. If boundary shapes are known, discrete edge points can be used to infer the curve parameters, allowing for accurate boundary reconstruction in the original image [43].

**Fig 13:**
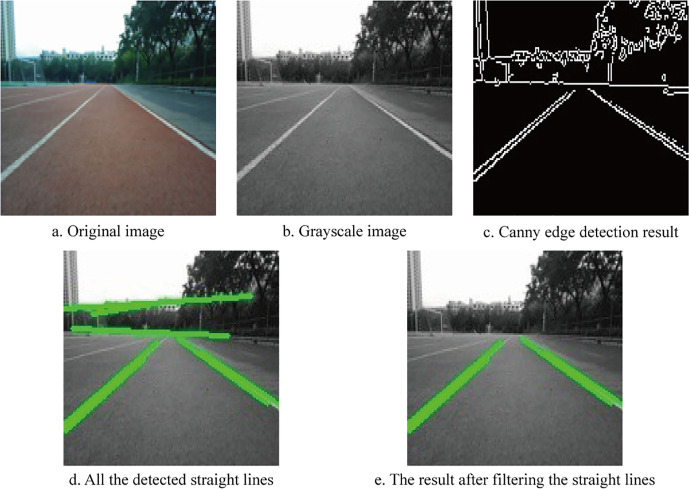
Example of runway marking.

**Fig 14:**
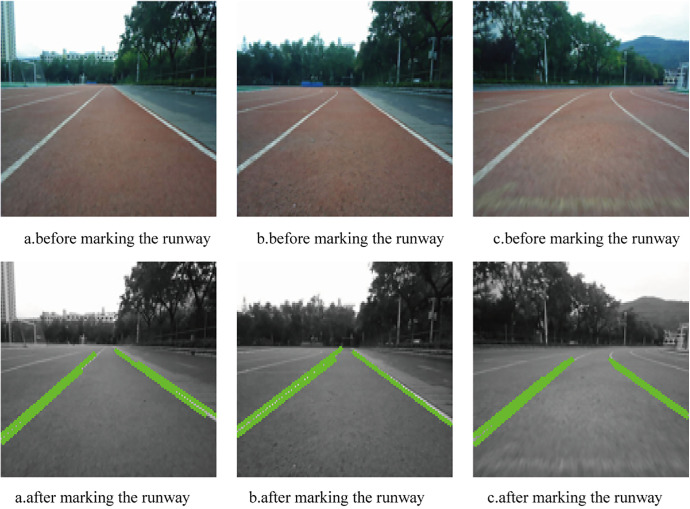
Comparison of the results before and after marking the runway.

To mark the runway in the training set images, the following processing steps are applied. First, the input image is converted into a grayscale image. Unlike color images, which typically have three channels (e.g., RGB), grayscale images contain only one intensity value per pixel. This conversion reduces the data volume by approximately two-thirds, significantly lowering the computational burden and accelerating subsequent processing. Next, the Canny edge detection algorithm is used to extract prominent edges from the grayscale image. The resulting edge map is then processed using the Hough Transform to detect straight lines. A green line is drawn over these detected lines to mark the runway boundaries. However, the presence of distant background objects such as trees or buildings can result in false detections that resemble runway edges. To minimize mislabeling, a filtering is introduced that retains only straight lines located in the lower half of the image, where the actual runway is more likely to appear. Fig 13 presents a step-by-step example of the runway marking process, while Fig 14 compares the images before and after enhancement.

This runway marking operation significantly improves the clarity of structural features. It enables the network to better learn the geometry and position of the runway while reducing the influence of irrelevant background elements. This enhancement improves the model’s ability to accurately identify runways under varying lighting conditions. When combined with other augmentation techniques such as contrast enhancement, chromaticity adjustment, and image flipping, this method further expands the diversity of the dataset and strengthens the model’s robustness and generalization capability.

### 5.4. Experimental environment and parameter configuration

The experiments were conducted on a Windows 10 system equipped with a 13th-generation Intel Core i5-13600F processor operating at a clock speed of 3.50 GHz, along with an NVIDIA GeForce RTX 4060 Ti graphics card (16GB of video memory). PyTorch version 1.12.1 was used as the deep learning framework, with Python as the programming language and PyCharm as the development environment. The selection of hyperparameters significantly influences the efficiency and convergence behavior of the model. After multiple rounds of tuning and optimization, the final hyperparameter settings were determined as follows: the learning rate was set to $\text{3}\times {\text{10}}^{-\text{4}}$. The batch size was adjusted to 32, and the number of training epochs to 100. The Adam optimizer was selected for gradient-based updates due to its proven effectiveness in deep learning tasks [44].

### 5.5. Model evaluation and performance comparison

#### 5.5.1. Evaluation indicators.

To evaluate the accuracy of the steering angle prediction network, the model’s outputs were compared with the ground truth steering angles generated by the robot. Three evaluation metrics were selected to assess performance comprehensively. The mean absolute error (MAE) quantifies the average angular deviation between predicted and actual values, offering insight into the overall driving smoothness. The mean square error (MSE) places more emphasis on larger deviations, making it a critical metric for identifying extreme errors that could lead to lane departure or collisions. The root mean square error (RMSE), derived as the square root of the MSE, offers a physically interpretable value in radians or angles, which facilitates comparison with acceptable real-world error thresholds. Together, these metrics provide a balanced assessment of average accuracy, sensitivity to outliers, and physical relevance.

The formulas used for these metrics are as follows. MAE is calculated as the average absolute difference between predicted and actual steering angles.


$$MAE=\frac{\text{1}}{n}\sum_{i=1}^{n}\left|y_{i}-\overset{\wedge }{y_{i}}\right|$$ (11)

MSE computes the average of the squared differences between the predictions and true values, calculated using the following formula:


$$MSE=\frac{\text{1}}{n}\sum_{i=1}^{n}{\left(y_{i}-\overset{\wedge }{y_{i}}\right)}^{2}$$ (12)

RMSE is the square root of the MSE and indicates the standard deviation of prediction errors. The calculation formula is:


$$RMSE=\sqrt{\frac{\text{1}}{n}{\sum_{i=1}^{n}\left(y_{i}-\overset{\wedge }{y_{i}}\right)}^{2}}$$ (13)

Among them $y_{i}$ is the actual steering angle, $\overset{\wedge }{y_{i}}$ is the predicted steering angle, and n is the sample size. In all three cases, smaller values indicate higher model accuracy. However, MSE and RMSE are more sensitive to outliers than MAE, which provides a more stable measure of average error.

#### 5.5.2. Performance comparison.

**Fig 15:**
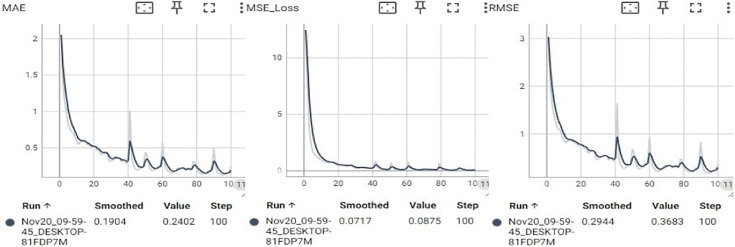
Basic ResNet18 network error curve.

**Fig 16:**
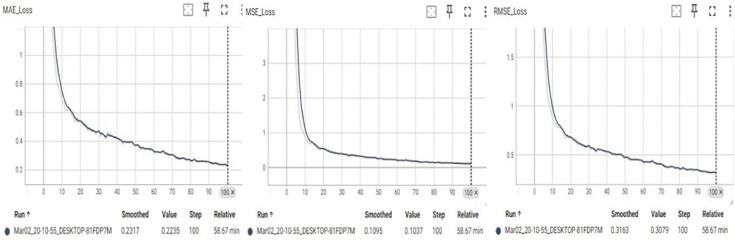
The error curve after improving the network.

To evaluate the predictive performance of the proposed network, comparative experiments were conducted using several baseline architectures, including ResNet18, ResNet34, ResNet50, MobileNetV2, and ShuffleNet. Each model was trained and tested on the same dataset under identical conditions. The results reported are averaged over multiple runs to minimize the influence of anomalous outcomes. Figs 15 and 16 illustrate the error curves for the baseline ResNet18 and the proposed improved model, respectively. Table 2 summarizes the evaluation metrics for each architecture.

**Table 2:** Evaluation metrics of the improved network and baseline models.

Model types	MAE	MSE	RMSE	Iteration time(h)
ResNet18	0.2492	0.0973	0.3801	2.212
ResNet34	0.2739	0.0912	0.4014	2.417
ResNet50	0.2733	0.0973	0.4009	2.506
MobileNetV2	0.2364	0.0983	0.3131	0.784
ShuffleNet	0.2482	0.1057	0.3244	0.687
New ResNet18	0.2281	0.1094	0.3108	0.998

From the data in Figs 15 and 16 and Table 2, the experimental results demonstrate that the improved network significantly enhances both the prediction accuracy and computational efficiency. Compared with the baseline ResNet18, the proposed model reduced training time by 54.88% and achieved reductions of 8.47% in MAE and 18.23% in RMSE. Moreover, the improved network exhibited more stable and smoother error curves throughout the training process. While MobileNetV2 and ShuffleNet benefit from efficient designs that leverage depthwise separable convolutions, their relatively limited capacity to represent complex features led to higher prediction errors in this task. This outcome confirms that the proposed improvements strike a favorable balance between computational efficiency and model accuracy.

To further examine the individual contributions of the Non-Local Block and Ghost Module, four ablation experiments were conducted. These included: ① the baseline ResNet18 network; ② ResNet18 with only the Non-Local Block; ③ ResNet18 with only the Ghost Module; ④ ResNet18 network with both modules integrated. The evaluation results of these experiments are presented in Table 3, confirming that each module independently improves the model and that their combined application yields the most substantial gains.

**Table 3:** Evaluation index results of ablation tests in each group.

Model types	MAE	MSE	RMSE	Iteration time(h)
①	0.2492	0.0973	0.3801	2.212
②	0.2478	0.0896	0.3874	2.471
③	0.2417	0.0873	0.3627	1.619
④	0.2281	0.1094	0.3108	0.998

The performance results from the ablation experiments reveal distinct contributions of the Non-Local Block and Ghost Module to the overall model performance. In the second group of experiments, the introduction of the Non-Local Block slightly improved prediction accuracy by enhancing the model’s ability to capture global contextual dependencies. As a result, the MAE, MSE, and RMSE values decreased modestly compared with the baseline model in the first group. However, this gain in accuracy came at the cost of increased computational complexity and longer iteration times. In contrast, the third group, which incorporated the Ghost Module, achieved a more substantial reduction in MAE, MSE, and RMSE. By effectively eliminating redundant features, this module improved the model’s computational efficiency while maintaining high prediction accuracy. Consequently, both the computational load and iteration time were significantly reduced relative to the baseline model. The fourth group combined both the Non-Local Block and Ghost Module, leveraging their complementary strengths to strike a balance between accuracy and efficiency. Although the Non-Local Block introduced additional computational overhead, the optimization effect of the Ghost Module sufficiently offset this cost. The resulting model achieved the best overall performance, demonstrating marked improvements in prediction accuracy and reducing iteration time compared with all other configurations.

## 6. Conclusion

This study presents a robot steering angle prediction network that integrates non-local attention and lane line guidance. Based on the ResNet baseline architecture, the model incorporates both the Non-Local Block and Ghost Module to enhance feature extraction from robot operating scenes. Additionally, a ReduceLROnPlateau learning rate scheduler is introduced to enable adaptive learning rate adjustment. Together, these enhancements significantly improve the network’s overall performance in three key areas:

(1) Network improvement and performance enhancement: The Non-Local Block strengthens the model’s capacity to capture global contextual information, while the Ghost Module reduces computational complexity by minimizing redundant features. Experimental results demonstrate that the improved network outperforms the ResNet18 baseline and other comparative models in both MAE and RMSE metrics, while substantially reducing iteration time.
(2) Module effectiveness verification: Ablation experiments confirm the individual and combined effectiveness of the Non-Local Block and Ghost Module. Their integration achieves the optimal balance between prediction accuracy and computational efficiency, validating the synergistic benefit of combining global attention with lightweight feature representation.
(3) Future work direction: Although the proposed model has achieved significant results, several areas merit further exploration. First, collecting data under more complex conditions, such as nighttime or low-visibility environments, could enhance generalization. Second, fine-tuning the parameter configurations of the Non-Local Block and Ghost Module and exploring additional lightweight modules may yield further gains. Finally, extending the model’s application to other complex robotic tasks could broaden its practical utility.

## Supporting information

S1 File Code files for reproducing the experiments. (ZIP)
